# The FEATURE framework for protein function annotation: modeling new functions, improving performance, and extending to novel applications

**DOI:** 10.1186/1471-2164-9-S2-S2

**Published:** 2008-09-16

**Authors:** Inbal Halperin, Dariya S Glazer, Shirley Wu, Russ B Altman

**Affiliations:** 1 Department of Genetics, 318 Campus Drive, Clark Center S240, Stanford, CA 94305, USA; 2 Program in Biomedical Informatics, MSOB X-215, 251 Campus Drive, Stanford, CA 94305, USA; 3 Department of Bioengineering, 318 Campus Drive, Clark Center S170, Stanford, CA 94305, USA

## Abstract

Structural genomics efforts contribute new protein structures that often lack significant sequence and fold similarity to known proteins. Traditional sequence and structure-based methods may not be sufficient to annotate the molecular functions of these structures. Techniques that combine structural and functional modeling can be valuable for functional annotation. FEATURE is a flexible framework for modeling and recognition of functional sites in macromolecular structures. Here, we present an overview of the main components of the FEATURE framework, and describe the recent developments in its use. These include automating training sets selection to increase functional coverage, coupling FEATURE to structural diversity generating methods such as molecular dynamics simulations and loop modeling methods to improve performance, and using FEATURE in large-scale modeling and structure determination efforts.

## Discussion

### Introduction: importance and overview

A central goal of molecular biology is to understand the functions of proteins, including their catalytic properties, binding sites, cofactors, interaction partners, and subcellular localization. Traditional experimental methods for function characterization cannot cope with the rate at which genomics efforts are generating data. Computational methods for function recognition require far less time and expense and so can augment experimental methods. Computational tools make it possible to query many proteins for many different functions at varying levels of specificity, from general enzymatic activity to binding sites.

Usually, computational methods require either the sequence or structure of the molecule of interest. One effective approach in sequence-based function prediction methods is to compare the known sequence to a collection of sequences whose functions are known, whether on a global or a local level. A high level of similarity found by such a comparison to an annotated sequence may allow the transfer of this annotation to the sequence of interest, based on presumed homology. BLAST [1] performs efficient sequence searches to facilitate such analyses. Searches within databases such as Pfam [2] and PROSITE [3], which contain models of short sequence motifs highly correlated with specific functions, may also allow function assignment based on sequence.

Inherently more important for the function of the molecule is its structure. The emergence of structural genomics (SG) has led to rapid advances in our knowledge of structure and structure determination. With the efficiency of structure determination methods now allowing high-throughput experiments [4,5], the number of structures available in the Protein Data Bank (PDB) [6] is providing a wealth of insight into structure-function relationships. Based on structures with known function, it should be possible to assign putative function to structures for which there exists no direct functional information. Annotation of molecular function by similarity is possible on the structural level as on the sequence level – by evaluating the similarity of global folds or local environments [7]. Structural similarity methods may employ chemical, physical, energetic or geometric criteria to recognize functional environments [8-10].

Many SG projects are targeting novel structures with low sequence identity to known proteins, in order to increase the ability to cover all fold families with at least one solved structure. Precise function can be reliably transferred only if sequence identity is at least 40% [11]; structure is significantly less conserved when sequence similarity is less than 50% [12]. As such, traditional sequence-based methods will not be enough to annotate a significant number of the novel protein structures being solved. Furthermore, with many of the proteins possessing novel folds, traditional global fold-based methods will also be less effective. Consequently, there is a need for structure-based methods that do not depend on global fold similarity or exact conservation of residues or residue geometry.

Our group is actively interested in structure based function prediction, and has, to this end, developed a robust function recognition algorithm called FEATURE, which examines 3D environments of molecules in a way that is neither strictly sequence nor fold based. FEATURE represents the local environments of a macromolecule using descriptors that capture chemical, physical and spatial features. In this article we provide an overview of the FEATURE framework for predicting protein function. In particular, we present recent efforts in improving and enhancing FEATURE's functional coverage and efficiency, and in applying FEATURE in novel ways.

### An overview of the FEATURE system

The FEATURE system can be broken down into three major components. The first is the way in which sites, or local protein microenvironments, are represented; the second part concerns model building and supervised machine learning methods; and the third involves site scoring and model evaluation. FEATURE is flexible in the sense that each of these three components is adaptable to the specific needs of an application.

#### Microenvironment representation

One of the most important aspects of any structure-based protein function modeling system is how information about a protein is represented and calculated. Protein structure information can be especially complex, so simplified abstractions are used to capture relevant features in a way that is computationally tractable. Methods such as CASTp [13] employ geometric abstractions to describe the shape, area, and volume of surface pockets and internal cavities, which are often correlated with functional sites. Geometry is also used to determine the relative position of several amino acids to each other as in 3D templates [14]. Other representations involve calculating values for physicochemical properties associated with locations or elements in the structure, such as solvent accessibility, hydrophobicity, electrostatic potential, the presence of residues or secondary structure, conservation or the presence of chemical groups [15-24]. Jambon *et al. *use a representation that combines both geometry and property-based components [25].

**Figure 1:**
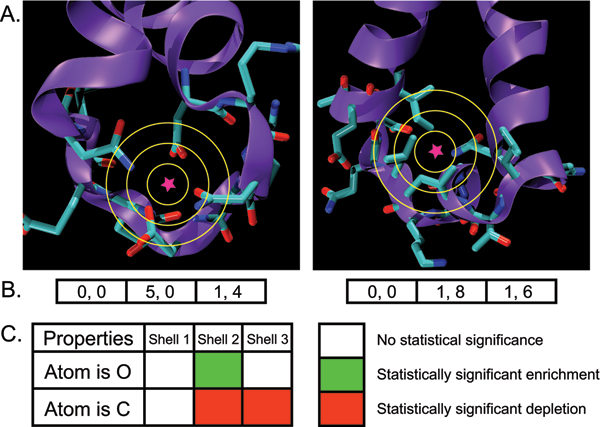
**Simplified example for building a FEATURE model**. **A. **An example of a positive site (left) and negative site (right), and their respective microenvironments. Properties are calculated in concentric spherical shells centered on each site (star symbol). **B. **FEATURE vectors calculated from the images in **A**, with oxygen atom count being the first property, and carbon atom count the second. The vectors are divided by shell for clarity. **C. **An example of a visualized FEATURE model is shown, based on the FEATURE vectors in **B**, and images in **A**. In Shell 2, oxygen atoms are more abundant in the positive site (5 counts) than in the negative site (1 count) and so oxygen atom count is considered a significantly enriched property in Shell 2 of the model. In contrast, carbon atom count is less abundant in the positive site (0 counts) compared to the negative site (8 counts), so carbon atom count is considered a significantly depleted property in Shell 2 of the model. In Shell 3, both the positive and the negative sites have 1 oxygen atom, so the model contains no significant difference for oxygen atom count in Shell 3.

FEATURE models a local protein microenvironment using a large number of physicochemical properties calculated at varying distances from the site (see Figure 1a for a simplified example). A site is defined as a 3D location in a protein structure, and its microenvironment is defined as a sphere centered on that location. In the typical use of FEATURE, 80 physicochemical properties (listed in Table 1) are computed in each of six 1.25 Å thick spherical shells – from 0 to 1.25, 1.25 to 2.5, 2.5 to 3.75, etc, up to 7.5 Å. Thus a FEATURE vector represents the site as a list of 480 values (see Figure 1b for a simplified example). The FEATURE method has also been tested successfully on other segmentations of volume, such as a cubic lattice [26,27].

**Table 1:** Physicochemical properties used by the FEATURE algorithm

**Atom – based**	**Residue – based**	**Secondary structure – based**
ATOM-TYPE-IS-C	RESIDUE_NAME_IS_ALA	SECONDARY_STRUCTURE1_IS_3HELIX
ATOM-TYPE-IS-CT	RESIDUE_NAME_IS_ARG	SECONDARY_STRUCTURE1_IS_4HELIX
ATOM-TYPE-IS-Ca	RESIDUE_NAME_IS_ASN	SECONDARY_STRUCTURE1_IS_5HELIX
ATOM-TYPE-IS-N	RESIDUE_NAME_IS_ASP	SECONDARY_STRUCTURE1_IS_BRIDGE
ATOM-TYPE-IS-N2	RESIDUE_NAME_IS_CYS	SECONDARY_STRUCTURE1_IS_STRAND
ATOM-TYPE-IS-N3	RESIDUE_NAME_IS_GLN	SECONDARY_STRUCTURE1_IS_TURN
ATOM-TYPE-IS-Na	RESIDUE_NAME_IS_GLU	SECONDARY_STRUCTURE1_IS_BEND
ATOM-TYPE-IS-O	RESIDUE_NAME_IS_GLY	SECONDARY_STRUCTURE1_IS_COIL
ATOM-TYPE-IS-O2	RESIDUE_NAME_IS_HIS	SECONDARY_STRUCTURE1_IS_HET
ATOM-TYPE-IS-OH	RESIDUE_NAME_IS_ILE	SECONDARY_STRUCTURE1_IS_UNKNOWN
ATOM-TYPE-IS-S	RESIDUE_NAME_IS_LEU	SECONDARY_STRUCTURE2_IS_HELIX
ATOM-TYPE-IS-SH	RESIDUE_NAME_IS_LYS	SECONDARY_STRUCTURE2_IS_BETA
ATOM-TYPE-IS-OTHER	RESIDUE_NAME_IS_MET	SECONDARY_STRUCTURE2_IS_COIL
ATOM-NAME-IS-ANY	RESIDUE_NAME_IS_PHE	SECONDARY_STRUCTURE2_IS_HET
ATOM-NAME-IS-C	RESIDUE_NAME_IS_PRO	SECONDARY_STRUCTURE2_IS_UNKNOWN
ATOM-NAME-IS-N	RESIDUE_NAME_IS_SER	
ATOM-NAME-IS-O	RESIDUE_NAME_IS_THR	
ATOM-NAME-IS-S	RESIDUE_NAME_IS_TRP	
ATOM-NAME-IS-OTHER	RESIDUE_NAME_IS_TYR	
HYDROXYL	RESIDUE_NAME_IS_VAL	
AMIDE	RESIDUE_NAME_IS_HOH	
AMINE	RESIDUE_NAME_IS_OTHER	
CARBONYL	CLASS1_IS_HYDROPHOBIC	
RING-SYSTEM	CLASS1_IS_CHARGED	
PEPTIDE	CLASS1_IS_POLAR	
	CLASS1_IS_UNKNOWN	
	CLASS2_IS_NONPOLAR	
	CLASS2_IS_POLAR	
	CLASS2_IS_BASIC	
	CLASS2_IS_ACIDIC	
	CLASS2_IS_UNKNOWN	
	PARTIAL-CHARGE	
	VDW-VOLUME	
	CHARGE	
	CHARGE-WITH-HIS	
	NEG-CHARGE	
	POS-CHARGE	
	HYDROPHOBICITY	
	MOBILITY	
	SOLVENT-ACCESSIBILITY	

In order to represent a local microenvironment, FEATURE determines the value of physicochemical properties in each of six concentric, spherical shells centered on the site of interest. Properties include those at the atom level, residue level, and secondary structure level.

The concentric spherical shells representation has both advantages and disadvantages. One disadvantage is that information about orientation and the relative position of atoms is discarded. However, discrete shells are favorable because they allow statistics to be gathered over the relevant volumes and calculation is relatively efficient, which allows FEATURE to serve as an initial filter for more expensive structure-based function prediction methods. Further advantages of this representation include unambiguous definition of a predicted site as a single point (i.e. Cartesian coordinates in the frame of the protein), accurate capture of properties of a cumulative nature such as partial charge, and the ability to change or add properties. The use of a single central point for each site means that models can be built with minimal prior knowledge of the geometry of the site – in other words, there is no need to establish other conserved points with which to define a non-spherical coordinate system. The use of spherical symmetry around this point also means that during search, each putative site center can be rapidly evaluated without the need to test alternative orientations around the point. Importantly, it allows identification of the physical and chemical features that are characteristic of functional sites, making the resulting models straightforward to interpret.

#### Model building by supervised machine learning

FEATURE uses supervised machine learning to combine significant properties into a model that can classify functional sites. In order to build a model, or description of a functional site, FEATURE requires two training sets. One consists of positive sites, which are 3D locations associated with positive examples of the function to be modeled; the other consists of negative sites, which are 3D locations not known to be associated with the function (see Figure 1a). Negative sites can be chosen manually or automatically by randomly sampling 3D locations of structures in the PDB with a similar range of atom densities compared to the positive sites. FEATURE vectors are calculated for each site in the training set.

Given a set of FEATURE vectors, a distribution of values can then be collected for each property in each shell (see Figure 1b). We determine whether a property is significantly overrepresented, significantly underrepresented, or not significantly different in positive sites relative to negative sites in a given shell by comparing the positive and negative training set distributions for the property in that shell. The significance of a property for distinguishing sites from negative sites is calculated over all properties in all shells, and naïve Bayes [28] is used to weight the properties most informative for distinguishing the positive and negative sites. FEATURE models are visualized using "fingerprints", which are color-coded grids that depict the significance of each property in each shell (see Figure 1c). It is critical to stress that the choice of negative sites defines the background distribution for all features and thus determines which features will be considered useful in identifying sites. Different models can result based on different strategies for defining the negative sites.

#### Site scoring and internal model evaluation

In order to determine performance statistics and score cutoffs for classification, the training sets are scored with the model, and sensitivity and specificity are estimated through k-fold cross-validation. Scores are calculated using a naïve Bayes scoring function, which operates on the assumption that the probability of a site belonging to a particular class is conditioned on the individual probabilities of observed, independent features. In the case of FEATURE, the features correspond to the physicochemical properties calculated in each shell, and their probabilities are derived from the training set distributions. A site's score is then the sum of the probabilities of obtaining an observed feature value given that the site is a positive site, taken over all significant features in the model. Score cutoffs are usually based on desired performance, and, as a default, are set to achieve 99% specificity on the training sets, as determined by cross-validation. In k-fold cross-validation, the training data is divided into k groups, and a model is trained on all but one of the groups and tested on the left out group.

Once a model is built and score cutoffs defined, potential sites can be scored using that model. FEATURE vectors are calculated for candidate sites in the same way as was done for training sites during model building, and scored using the same naïve Bayes scoring function. The resulting scores indicate the likelihood that the potential site is a positive site, depending on the score cutoff for that model. When available, the validity of every new model is assessed with an independent test set [18-20].

#### FEATURE in practice: workflow, training set selection, and manually-curated models

**Figure 2:**
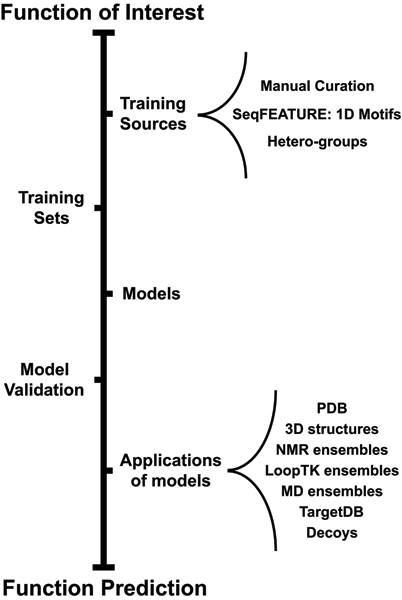
**FEATURE framework overview**. The outline of the steps necessary to predict a possible function for a protein is illustrated. In order to build a FEATURE model, one must first define the function of interest and create positive and negative training sets from the appropriate data sources. Then, the model is trained and evaluated on the training sets. The validated model can be used for function prediction. Certain steps in the outline, such as extracting training sets and model building are straightforward, as described in section "An overview of the FEATURE system". Other steps, such as determination of data sources for training sites and application of models, are more flexible. For example, training sites may be derived manually or automatically selected using annotated hetero-groups or sequence motifs. In addition, the resulting models can be applied towards static structures from the PDB or structure prediction decoys, or for dynamic function prediction over ensembles of structures generated using molecular dynamics simulation.

Creating a new model involves a typical workflow (see Figure 2) that begins by choosing a function of interest and defining a biologically reasonable definition of the Cartesian center point for that function (e.g. the central position in a binding site or the position of a key atom in an active site). Positive and negative training sets are then created and used to train the model. Cross-validation of the model on the training sets allows definition of score cutoffs based on desired performance, and whenever an independent test set is available, model performance can be further assessed. Once a model is built and a score cutoff has been defined, FEATURE can predict functional sites in structures of interest.

An especially important step in model training is the selection of sites for the positive training set, and, in order to tune performance, the negative training set. The first FEATURE models were manually curated in that the positive and negative training sets were built and verified by hand using published literature. These include calcium-binding [18] and ATP-binding [19] site models. The calcium-binding model has especially good performance, and is currently being used in multiple ongoing projects to expand FEATURE's capabilities and applicability, described later in this overview. Our recently published zinc-binding model [29] is the best performing zinc binding predictor currently available. We have also applied FEATURE to function prediction in RNA structures with two magnesium binding models, one for diffuse binding and one for site-specific binding [30].

From its manually-curated beginnings, FEATURE has expanded to include automatic generation of training sets using sequence motifs, PDB annotations, and even a clustering of FEATURE vectors encompassing a non-redundant subset of the entire PDB. Functional coverage by the FEATURE system is enhanced when we employ multiple and diverse strategies for site selection. We describe our current work in the area of site selection in more detail below.

### Increasing functional coverage

While having a highly specialized and performance-tuned model for recognizing a particular function is extremely valuable, it is becoming increasingly important to have wide coverage of protein function space. SG initiatives are causing a rapid expansion in the numbers of uncharacterized protein structures, many with very low sequence or even structural similarity to known proteins [31]. In order to expedite the annotation of structurally novel proteins, we need good and varied structure-based models of function. Structure-based models may also highlight heretofore unappreciated but interesting regions in partially characterized poly-functional proteins. Within the FEATURE framework, we have developed several strategies for expanding functional coverage.

#### SeqFEATURE – transforming 1D motifs into 3D models

Protein sequence data is extremely useful for deducing information about a protein's structure, interactions and function. Given its ubiquity, it comes as no surprise that there are numerous tools for recognizing function based on sequence. Pfam, Panther [32], PROSITE, and Superfamily [33] are just a few of the publicly available databases and methods for characterizing protein families or functions; many of them are conglomerated into single integrated tools like InterProScan [34] and ProFunc [8,10].

Most of the tools perform very well under most circumstances, but pattern matching tools such as PROSITE can be prone to false predictions and even the best tools, usually employing Hidden Markov Models, can be rendered less effective when sequence identity to known proteins is less than 30% [35]. 3D models have the potential to overcome this limitation, and can support a broader range of applications such as loop modeling and folding (see sections "Loop modeling" and "Decoy filtering").

In order to enhance both FEATURE's functional coverage and the performance of 1D motifs, we developed an extension to FEATURE, called the SeqFEATURE, that transforms sequence-based models into structure-based ones [20,35]. Given a 1D motif, SeqFEATURE algorithm automatically extracts structures from the PDB that contain the motif to form a positive training set. One parameter that must be determined is the site center for each model. In the case of a 1D pattern, the center might be a functional atom on a functional residue contained in the pattern. SeqFEATURE finds all such 3D examples in a non-redundant subset of the PDB to be used as a positive training set. When a pattern contains more than one functional atom, multiple models are built centered on each one. The overlapping models can be used singly or in concert to predict the functional site.

Recently, we have applied SeqFEATURE to 44 regular expression patterns from the PROSITE database of functional motifs to produce a library of 136 automatically derived and trained models [35] (see section "Availability"). The models exhibit a wide range of performance; however, over three-quarters of them have an area under the curve (AUC) greater than 0.8 based on cross-validation. Further analysis using a test set derived from manually curated true positives, false positives, and false negatives for each PROSITE pattern showed that the models did not always detect all of the true positives, but they almost always made fewer false positive and false negative predictions than PROSITE.

In a comparison against some of the leading sequence and structure-based function prediction methods, the SeqFEATURE library performed competitively. When the sequence identity and structural similarity of the test set proteins to the training set proteins was reduced, however, the SeqFEATURE library demonstrated a marked robustness that was not matched by any of the other methods. FEATURE's independence from specific sequence and structure elements allows it to perform with greater sensitivity on novel or unique proteins than other methods that rely on conservation.

In principle, SeqFEATURE can be applied to build models for other sequence-oriented motif databases, such as Pfam or PRINTS [36], to generate many more functional site models quickly and automatically, greatly increasing FEATURE's coverage of protein function space. In addition, the enhanced performance at low sequence identity makes FEATURE a particularly relevant method for aiding the annotation of novel protein structures.

#### Hetero-groups-based functional site models

Many proteins and nucleic acid molecules require small molecular ligands or cofactors such as ATP or NAD in order to function properly. Ligands and cofactors, generally referred to as 'hetero-groups', are diverse. There are currently 7,642 types of hetero-groups in the PDB. These hetero-groups appear in as many as 76.6% of structures in this database. The prevalence of these hetero-groups among biological macromolecules makes them good candidates for automatic training of functional models using FEATURE.

The process of building a hetero-group-based model follows the guidelines described in section "An overview of the FEATURE system". A positive training set for a given hetero-group begins with collection of protein structures containing this hetero-group, namely holo structures. There are many databases of ligand-binding structures, including PDBSum [37], Relibase [38], Hic-Up [39], PLD [40], and PDB-Ligand [41]. The proteins that a given ligand binds are often homologous and present the same binding structure to the ligand. However, there are also many instances wherein a given ligand binds to the same or homologous protein in different binding environments. Therefore, representative structure selection among homologous proteins should be carefully executed. Some of the databases allow automatic superimposition of binding sites and sequence identity filtering which is necessary for representative selection. Once a non-redundant set of holo proteins is composed it may not have a sufficient number of structures. A minimum of five representative structures is required for a positive training set for FEATURE. Since larger datasets are more favorable, apo structures, determined without a hetero group, can supplement the datasets.

Automatic training of hetero-group based models presents us with many challenges. One major challenge is choosing the model center. An obvious strategy is to use the centroid of the ligand as a center; however, this choice sometimes results in poor performance. Another option is to center on active atoms, but these need to be manually curated for the most part. The larger hetero-groups – containing as many as 390 atoms (e.g. RNA) – present another challenge, as they cannot be fully described within FEATURE's 'traditional' shell size of 7.5 Angstroms. The shell size can be enlarged only to some extent without altering the signal derived from accumulating properties of atoms within shells.

**Figure 3:**
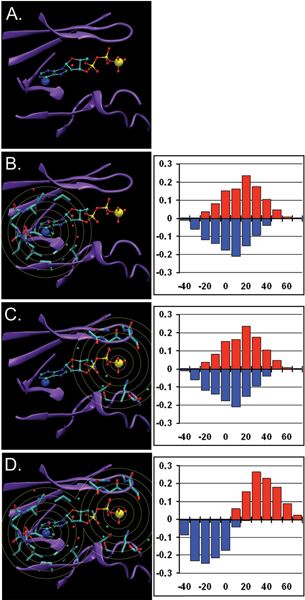
**Illustration of the potential value of combining FEATURE models**. **A. **An ATP binding pocket in PDB structure 1CSN. Enlarged are N6 (blue) and PG (yellow) atoms in ATP. **B. **Parts of the molecule considered by a putative FEATURE model centered on N6 with shells out to 7.5 Å. Such a model might have poor ability to separate positive sites and negative sites, as shown in the histogram on the right with substantial overlap of (red) positive sites and (blue) negative sites. **C. **Parts of the molecule considered by a putative FEATURE model centred on PG with shells out to 7.5 Å. Again, such a model might have poor discriminating ability, as shown in the score distributions on the right for (red) positive sites and (blue) negative sites. **D. **Parts of the molecule considered by an analysis which combines the two marginal models in **B **and **C**. By evaluating hits to multiple models along with appropriate distance constraints, it is possible to achieve better combined performance than either single model alone, as show in the putative plot on the right.

A better strategy is to build several 'sub-models' for different parts of the hetero-group and to combine them into a single model using a range of distances between model centers (see Figure 3). This approach increases the complexity of model building significantly because sub-models can be applied jointly in a combinatory fashion. Preliminary results for ATP-binding site prediction using a two-center approach suggest, however, that performance does improve with the addition of even one more center.

#### Clustering the PDB to discover and annotate new structural motifs

One limitation of the site selection strategies described in sections "SeqFEATURE – transforming 1D motifs into 3D models" and "Hetero-groups-based functional site models") is that they depend on existing annotation and cannot be used to discover new functions or potentially interesting structural motifs. To overcome this, we calculated FEATURE vectors for all residues in a non-redundant subset of the PDB – approximately 2 million vectors in all – and clustered them to reveal groups of residues sharing similar microenvironments [42]. In order to make calculation on this scale feasible, features were converted to binary values with minimal reduction in clustering accuracy. A number of the clusters corroborate with known PROSITE motifs, indicating that this strategy can reveal truly interesting groups of sites that may be used to construct new FEATURE models.

Although the capability to discover new motifs is important, its value is diminished unless there is a description of the possible biological or functional roles a new motif may have. One way to alleviate this problem is by generating descriptive text for each cluster automatically. Methods that address similar problems [43-45] rely, for the most part, on standard vocabularies such as the Gene Ontology [46], which are organized at a higher level of conceptual granularity than raw text. While the use of controlled terminologies can resolve many of the challenges surrounding text mining, processing the raw text may reveal less obvious connections. Such an approach could prove useful not only for characterizing clusters of similar protein microenvironments, but also for clusters or lists of any biological entities that have an associated literature, such as genes, drugs, or diseases.

Preliminary studies on test clusters of proteins derived from PROSITE motifs using a simple entropy-based scoring function demonstrate that this approach is able to detect the fundamental molecular function shared by the members of the cluster (i.e. the PROSITE motif) in addition to more detailed information, such as active site residues (see Table 2).

**Table 2:** A text mining approach using an entropy-based scoring function rediscovers the molecular function of proteins sharing PROSITE motifs

**Motif # of proteins # of documents**	**Terms**
**EF_HAND**	ef-hand
36	calcium-bind
183	calcium
	ca 2+
	calcium-bind protein
	ca
	2+ bind
	2+
	ef-hand motif
	calmodulin

**TRYSIN_SER**	serin proteinas
11	proteinas
108	chymotrypsin
	serin
	serin proteas
	elastase
	ser-195
	his-57
	proteinas especially
	proteolyt

**PROTEIN KINASE_ST**	protein kinas
15	catalyt domain
107	phosphoryl
	substrat
	autophosphoryl
	phosphoryl site
	kinas
	threonin
	catalyt
	constitutively active

The method extracts text from the abstracts of references annotated in each protein's Swiss-Prot record, pre-processes the text (tokenization into terms, removal of non-content words, and basic stemming to normalize word forms), and scores terms based on their distribution across proteins and their relative significance in the entire corpus of Swiss-Prot referenced documents. With no additional normalization, concept and word redundancy may be observed. Although still very preliminary, the method is able to capture the molecular function for each cluster of proteins shown: "ef-hand" and "calcium binding" for EF_HAND; "serine proteinase", "proteolysis", and the active site residues "ser-195" and "his-57" for TRYPSIN_SER; and "protein kinase", "phosphorylation", "catalytic domain" and the substrate residue "threonine" for PROTEIN_KINASE_ST.

### Improving FEATURE's performance

Extended functional coverage improves the FEATURE framework with respect to the functional space that can be explored. Additionally, it is possible to improve the ability of FEATURE to recognize functional sites, for example, by exploring the conformational space of the molecules in question. In order to perform their function, most proteins undergo dynamic changes within the active site. Methods that use static structures to predict function do not take structural dynamics into account. However, as the number of solved static structures increases in the PDB and the performance of static methods does not reach desirable levels, the importance of sampling the conformational space of the molecules becomes more apparent.

#### Dynamics improves efficiency of function annotation methods based on structure

The methods we have reviewed above generally rely on analysis of static structures solved by X-ray crystallography and Nuclear Magnetic Resonance (NMR) techniques. Both techniques, however, have characteristics that may preclude structure-based function prediction methods from performing at the highest levels of sensitivity. In X-ray crystallography, crystal packing may effectively rigidify proteins into compact conformations, which may not represent good averages of the conformational space of the molecules in solution. In order to overcome this limitation, time-resolved X-ray crystallography allows determination of many conformations at 1 picosecond intervals. Using this technique, Schotte *et al. *observed nuances of the inner workings of a myoglobin mutant as it progressed from a carboxy to a deoxy state [47]. However, time-resolved X-ray crystallography is not currently amenable to application in a high-throughput manner, since it requires molecules to be photosensitive, and data interpretation can be nontrivial [48]. These experiments illustrate that it is necessary to take into account the dynamic nature of molecules in order to understand its functional space.

**Figure 4:**
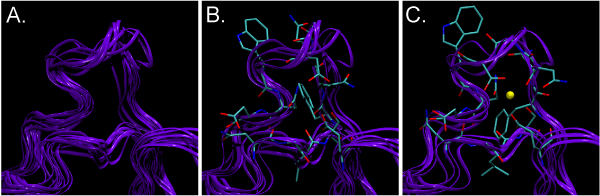
**NMR ensemble scanning results for PDB structure **2B1O. 2B1O is a structure of a protein which is known to bind calcium (Ca^2+^). The NMR ensemble for 2B1O contains different conformations of the structure, some of which show different proclivities for binding Ca^2+^. **A **shows 10 NMR generated structures for one of the known Ca^2+ ^binding loops, superimposed to minimize RMSD; **B **shows loops that FEATURE does not identify as Ca^2+ ^binding, corresponding to NMR models 1, 3, 4, 5, 6, and 10; and **C **shows loops that FEATURE does identify as Ca^2+ ^binding, corresponding to NMR models 2, 7, 8, and 9. In **B **and **C**, sidechains in the vicinity of the FEATURE hits are shown for the highest scoring NMR model (score ~39 for **B **and ~64 for **C**). In **C**, one of the hits that scored over the model threshold of 50 is shown as a yellow ball. Notice the differences in the conformations between side chains in **B **and **C**: the entire loop is wider in **C**, and coordinating oxygens form a ring around the hit, while in **B **they are more scattered. There is also a difference in the conformation of phenylalanine ring, which essentially blocks the Ca^2+ ^binding spot in **B **but is rotated away from the site to allow possible Ca^2+ ^binding in **C**.

Although NMR structures do not generally achieve the resolution of structures solved by X-ray crystallography, they better represent the conformational space of the molecules because they typically produce an ensemble of structures. Since the molecules are all in solution during the NMR procedure, this ensemble of structures provides an opportunity to understand the dynamic behavior of molecules. Recent studies highlight the value of the structural diversity contained in the NMR ensembles. We examined several such ensembles (see Table 3) with a FEATURE Ca^2+ ^binding model [19]. A subset of structures from most ensembles revealed Ca^2+ ^binding sites (see Figure 4). The fact that all the structures did not exhibit Ca^2+ ^binding behavior is noteworthy, because it demonstrates that the dynamics may influence our ability to recognize function.

**Table 3:** Results of NMR ensembles scanned with FEATURE Ca^2+ ^binding site model

**Protein Name**	**PDB ID**	**Number of Models**	**Number of Models Characterized as Calcium Binding**
Lipoprotein receptor-related protein repeat 8	1CR8	20	20
Lipoprotein receptor-related protein repeat 3	1D2L	20	20
RALBP1-intercating protein	1IQ3	18	18
Rous Sarcoma virus receptor	1JRF	20	20
Tyrosine-protein kinase SRC	1KSW	20	20
Calerythrin	1NYA	20	20
Human Notch1	1PB5	16	16
Porcine pancreas phospholipase A2	1SFW	18	1
Rational design of a calcium-binding adhesion protein	1T6W	20	3
Human beta parvalbumin	1TTX	20	20
Cytochrome c peroxidase *	2B10	10	4
Matrilysin	2DDY	25	24
Calcium-binding protein p22	2E+30	20	17
Sodium/calcium exchanger 1 domain 1	2FWS	20	18
Sodium/calcium exchanger 1 domain 2	2FWU	20	18
Rat megalin	2I1P	20	19
Relaxin receptor 1	2JM4	24	22
Yeast frequenin	2JU0	15	15

Scanning of the 18 NMR ensembles with the Ca^2+ ^binding model revealed structural heterogeneity among the structures in the ensembles. In several, most of the models exhibited Ca^2+ ^binding conformations, while in others, only a few. The first and the second columns contain names and PDB IDs of the examined proteins, respectively. The third and fourth columns show the total number of models and how many of those were identified by FEATURE as Ca^2+ ^binding in the NMR ensemble, respectively. * – Results of this scan are shown in Figure 4.

Computational methods allow us to explore the dynamics of molecules on the scales that are not experimentally accessible while assessing their potential functions [49]. In particular, molecular dynamics (MD) simulations provide large ensembles of structures. Recent work demonstrates that MD simulations generate structural diversity useful for the assignment of function. Eyrisch *et al. *used MD simulations to improve efficiency of predicting functional surface pockets, which may be obscured in static PDB structures [50]. In the pharmaceutical industry, improvement in the prediction of functional pockets may assist in the development of more efficient drugs. Frembgen-Kesner *et al. *showed that cryptic drug binding sites, which appear only when the target has bound a ligand already, become more apparent over the course of MD simulations than in the original PDB structures [51].

We demonstrated the value of examining structural diversity generated by MD simulations with FEATURE to identify Ca^2+ ^binding sites [52]. In the case of parvalbumin β, results of FEATURE coupled with dynamics recapitulated the behavior of the protein's Ca^2+ ^binding sites with and without synthetic mutations (PDB IDs 1B8C and 1B9A, respectively). Further experiments are underway to establish the extent to which sampling conformational diversity with MD simulations improves efficiency of functional predictions made by FEATURE. Functions other than Ca^2+ ^binding may be explored with various FEATURE models or alternative structure-based function prediction methods by evaluating MD ensembles of structures.

#### Loop modeling

Although SG initiatives are accelerating biological structure determination, it still lags behind the production of new genomic sequences. Roughly a third of all protein sequences can be modeled based on similarity to a known three-dimensional structure, but one of the major limiting factors is the ability to model structurally variable loop regions [53]. Loops participate in many active and binding sites in proteins. *A priori *knowledge of a loop's function can potentially be used to limit its conformational space, thereby assisting in achieving a more accurate ensemble. Such knowledge can result from sequence-based or structure-based predictions or from experiments.

In order to explore FEATURE's utility in loop modeling, loop conformations were generated by two methods: seed sampling and deformation sampling [54]. Both methods satisfy constraints on kinematic closure and clash avoidance. Seed sampling generates structurally diverse loop, whereas deformation sampling explores a more limited region close to the provided starting conformation. We examined the ability of these methods to generate 'functional' loops conformations that are similar to the native structure and could be recognized by FEATURE. Calcium binding loops of parvalbumin (1B8C, Ala51-Ile58) and grancalcin (1K94, Ala62-Asp69) were modeled with seed sampling and deformation sampling respectively. Both routines were able to build at least one functional loop, as evaluated by FEATURE, within a ~100,000 conformation ensemble.

Increasing the accuracy of loop conformation prediction using FEATURE as a filter for functionally plausible conformations can be applied not only to homology modeling but also to the task of modeling missing loops in experimentally-derived structures. Since loops tend to participate in ligand binding, dimer formation and enzymatic activity, they are an essential part of the structure and may hold clues to the elusive structure-function relationship. We are currently validating this method on a dataset of existing loops in order to predict missing functional loops reliably.

### Extending FEATURE to new applications

The flexibility of the FEATURE framework has proven to be extremely useful for increasing FEATURE's functional coverage and improving not only individual FEATURE models, but also the performance of methods solving slightly different problems, such as loop modeling. Here, we describe some novel applications of FEATURE that have broadened its utility.

#### Structural genomics and scanning for function in high-throughput

Structures solved by SG projects often bear little resemblance to known proteins in either sequence or structure, making annotation especially challenging. Previously, we showed that the sensitivity of the SeqFEATURE library of automatically derived functional site models (described in section "Increasing functional coverage") is more robust than that of some of the leading sequence and structure-based function prediction methods when sequence identity and structural similarity to known proteins are low [35]. As a result, the SeqFEATURE models should be valuable for suggesting potential functions for novel SG targets.

With this in mind, we scanned all of the SG targets in TargetDB [55] associated with unknown function through October 2007 using the SeqFEATURE library, filtered for the highest confidence predictions (based on model-dependent score cutoffs), and compared them to predictions made by a number of popular sequence and structure-based methods [35]. For a substantial fraction of these targets, the sequence-based methods made no significant predictions; for a smaller fraction, the structure-based methods had no or low confidence predictions as well. Those targets for which SeqFEATURE made a high confidence prediction but other methods did not are compelling candidates for further study (see section "Availability").

In keeping with the need for high throughput, we have also scanned the entire PDB (up to February 2006, about 35,000 proteins) with the entire SeqFEATURE library (see section "Availability"). The scan took about one day to complete on 13 parallel processors, suggesting that a large-scale scan of many structures with many functional site models is actually quite efficient. With the structure determination pipeline improving and novel protein structures increasing every year, scanning for function in a high-throughput fashion will become a necessary enterprise.

#### Decoy filtering

One of the major goals of three-dimensional (3D) structure prediction methods, such as comparative modeling, threading and *ab initio *folding, is to elucidate function from a 3D structure. Determining the occurrence and location of active and binding sites within a structure helps achieve this goal. In 1999, Wei *et al. *predicted two calcium-binding sites in model structures, or decoys, of a vitamin D-dependent protein [56]. These decoys, generated by Park and coworkers [57,58], include near native structures. Root mean squared deviation (RMSD), which measures pairwise structural similarity, ranged from 0.95 Å to 9.39 Å between the decoys and the native structure.

Despite the existence of near native decoys, the quality of the calcium-binding microenvironments had only a very weak correlation with the overall RMSD. Moreover, the correlation between 'local RMSD' and FEATURE scores was also weak. Only when the quality of the local structural neighborhood around the calcium site is high does the modeling of the binding sites become reliable. Perturbation of atoms' positions within the native structure generated 100 decoys with a local RMSD of 0 to 1.7 Å [56]. The RMSD of these structures correlated with FEATURE's ability to recognize the functional site.

Recently we re-examined decoy selection with FEATURE. Current improved methodologies for *ab initio *folding are able to generate decoys similar in quality to the previously used perturbed structures. Some small proteins (under 100 amino acids) can be refined up to a near-atomic resolution level [59]. Using FEATURE, we scanned five hundred low scoring decoys for twelve calcium-binding proteins generated with Rosetta [60]. FEATURE scores were able to reduce the number of decoys while enriching for near-native conformations, sometimes with improvements of the average RMSD to known crystal structure moving from 9 to 5 Angstroms (Das Rhiju and Halperin Inbal, unpublished results).

These preliminary results support the potential value of incorporating FEATURE into the *ab initio *folding scheme. Much of the calculation time in *ab initio *folding is spent on the side chain packing of the different main chain conformations generated in the main chain optimization stage. The ability to reduce the number of main chain conformations after this stage while keeping most of the correct conformations would be highly valuable for lowering the computational cost.

### Availability

FEATURE models, data, and source code are available online for public use. The WebFEATURE website [61,62] allows functional scans of PDB structures using any of the manually curated models or the models in the SeqFEATURE library, as well as the option to scan using the entire SeqFEATURE library. The improved zinc binding model is also available for scanning [63]. Single SeqFEATURE model scans require only a few seconds to run, scanning with the entire SeqFEATURE library may take about a minute, and manually curated models may take varying lengths of time depending on the size of the input structure. Job status notification can occur either interactively on the website or through email notifications, and results can be interactively viewed in a web browser.

Data from the PDB scan and high-confidence predictions for TargetDB structures can be downloaded from the "Data" section of the WebFEATURE site [64]. Source code for FEATURE is accessible from SimTK [65], a repository for biological structure software maintained by the SimBIOS Center for Biomedical Computation [66,67]. FEATURE has been downloaded about 150 times since being made available on SimTK. In addition, WebFEATURE is currently seeing almost 2,500 unique visitors a month.

## Conclusion

FEATURE is a powerful function recognition framework that has been adapted to new paradigms in function annotation and structure modeling. Importantly for the annotation of structural genomics targets, FEATURE robustly models molecular functions without relying on significant sequence or fold similarity. Creating training sets automatically from many different sources and discovering new functions through unsupervised clustering of microenvironments improves functional coverage. Function annotation approaches that recognize and treat the dynamic nature of molecules as essential are proving to be more successful than their static counterparts, and FEATURE can be easily coupled to simulations to enhance function recognition. Structure determination and loop modeling efforts also benefit from the addition of FEATURE as a filter. As structural genomics and structure determination efforts advance and evolve, structure-based modeling will become more important. FEATURE is uniquely poised to take advantage of and assist in these efforts.

## Abbreviations

SG = Structural Genomics. PDB = Protein Data Bank. AUC = Area Under the Curve. NMR = Nuclear Magnetic Resonance. PSSM = Position Specific Scoring Matrix. RMSD = Root Mean Squared Deviation

## Competing interests

The authors declare that they have no competing interests.

## Authors' contributions

IH, DSG, and SW wrote the manuscript and carried out research described therein. RBA conceived and edited the manuscript.
